# Efficient Mechanochemical Bifunctional Nanocatalysts for the Conversion of Isoeugenol to Vanillin

**DOI:** 10.3389/fchem.2018.00077

**Published:** 2018-04-03

**Authors:** Somayeh Ostovar, Ana Franco, Alain R. Puente-Santiago, María Pinilla-de Dios, Daily Rodríguez-Padrón, Hamid R. Shaterian, Rafael Luque

**Affiliations:** ^1^Department of Chemistry, University of Sistan and Baluchestan, Zahedan, Iran; ^2^Grupo FQM-383, Departamento de Química Orgánica, Universidad de Cordoba, Cordoba, Spain; ^3^Peoples Friendship University of Russia, Moscow, Russia

**Keywords:** vanillin, nanocatalyst, catalytic oxidation, sulfonic groups, SBA-15

## Abstract

A bifunctional nanocatalyst composed of iron containing SBA-15 material modified with sulfonic acid groups was synthesized by a mechanochemical approach. A full characterization of the obtained nanocatalyst was performed by N_2_ physisorption isotherms analysis, transmission electron microscopy (TEM), X-ray powder diffraction (XRD) and Fourier-Infrared Spectroscopy (FT-IR). The mechanochemically synthesized nanocatalyst displays a high isoeugenol conversion to vanillin under mild conditions using H_2_O_2_ as oxidizing agent. Interestingly, this conversion resulted to be higher than that one obtained with the same material synthesized by an impregnation method. Additionally, the nanocatalyst showed excellent reusability over four successive runs under the studied reaction conditions.

## Introduction

Nowadays, in the field of Green Chemistry, the conversion of lignocellulosic biomass into value-added chemicals has become a challenging topic in both academic and industrial research areas (Zakzeski et al., 2010; Xu et al., 2014; Behling et al., 2016). Lignocellulosic biomass is composed by three major components (cellulose, hemicelluloses, and lignin), which offers specific opportunities to produce a myriad of valuable chemicals (Rinaldi et al., 2016). Among them, the highly functionalized and aromatic structure of lignin facilitates the designing of desired chemical platforms. In this sense, derived compounds of lignin such as eugenol, isoeugenol, and ferulic acid have been employed to obtain vanillin through a simple oxidation route (Gusevskaya et al., 2012). Vanillin (4-hydroxy-3-methoxybenzaldehyde) possesses a number of valuable applications for food, beverages, perfumery and pharmaceutical industries owed to it represent the principal flavor and aroma component in vanilla. Currently, the major amount of vanillin is obtained from petro-based compounds, especially guaiacol and glyoxylic acid by non-environmental friendly synthetic routes such as Riedel process, Huang et al. (2013) attaining low qualities of the final product. To overcome these drawbacks, greener strategies based on the catalytic oxidation of lignin model compounds have been developed using metal functionalized mesoporous silica material as efficient nanocatalysts (Augugliaro et al., 2012; Franco et al., 2017).

Noteworthy, it have been reported that the anchorage of organic functional moieties like –COOH and -SH can interact with silanol groups of the mesoporous silica framework and control its surfaces properties and consequently its catalytic performance (Li and Yan, 2010; Rajabi et al., 2011). Among various functionalized groups, sulfonic acid group cause a remarkable increase on the catalytic performance of the catalysts due to its favored interactions with the metal active sites and its surface acid properties (Akiyama et al., 2011). Incorporation of sulfonic groups on the surface of ordered mesoporous such as SBA-15 and MCM-41 is performed generally by two step process (Kapoor et al., 2008): co-condensation or anchoring of -SH containing alkoxide precursors and the subsequence oxidation in the present of H_2_O_2_, which are somewhat complicate and incomplete routes (Melero et al., 2006). In previous works, the lack of order in few domains of sulfonic groups at the pore surfaces have decreased the catalytic yields caused by the aggregation of nanoparticles on the outer surface of the nanocatalysts (Kim et al., 2009; Jackson et al., 2013). For these reasons, the designing of novel efficient synthetic routes toward the synthesis of high performance nanocatalysts composed of metal containing mesoporous silica modified with sulfonic acids groups is still a challenge.

**Figure 1:**
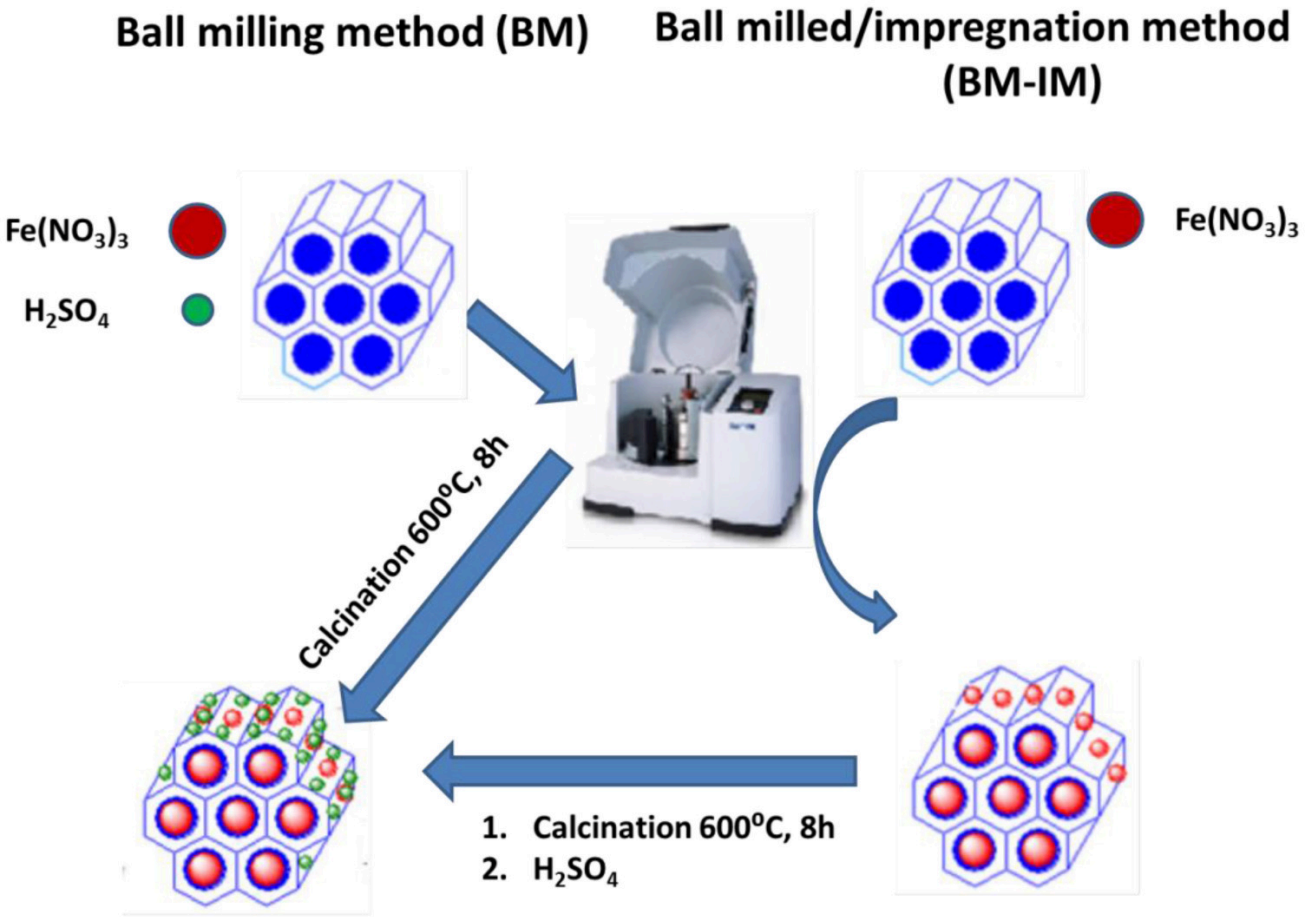
Schematic illustration of the synthetic strategies of the two nanocatalysts.

In this work, we propose an unprecedented one-pot mechanochemical synthesis of bifunctional nanocatalysts (Figure 1) based on iron containing SBA-15 functionalized with sulfonic groups (Fe-SBA15-HS${\text{O}}_{3}^{\text{BM}}$) for the selective oxidation of isoeugenol to vanillin. The methodology used represents a greener and innovative route to construct highly functionalized mesoporous silica nanomaterials taking advantage of their nanochannels with controllable pore size, high surface area and tunable reactivity. Also, we have compared the catalytic performance of the novel nanocatalysts with others synthesized by conventional methods such as the ball milled/ impregnation method (Fe-SBA15-HS${\text{O}}_{3}^{\text{BM}-\text{IM}}$).

## Experimental

### Materials

All chemicals chloride were obtained from Sigma–Aldrich with pure analytical degree.

### Synthesis of SBA-15

The ordered mesoporous silica SBA-15 was synthesized following a procedure described in the literature (Jarry et al., 2006). The triblock copolymer Pluronic P123 (0.41 mmol) was dissolved in a HCl aqueous solution (2M) at 35°C. Subsequently, Tetraethyl ortho-silicate (TEOS) (25 mmol) was added drop wise to the solution mentioned above. The resulted solution was agitated for 24 h at 35°C. After that the mixture was subjected to a hydrothermal treatment in an oven at 100°C for 48 h. The resulting material was filtered and dried at 60°C. Finally, the template was calcined at 550°C for 8 h to remove it.

### Preparation of FE-SBA-HS${\text{O}}_{3}^{\text{BM}}$ nanocatalysts

Fe-SBA-HSO_3_^BM^ catalyst was prepared using 0.5 g of SBA-15 as silica support, 1.34 g of Fe(NO_3_)_3_·9H_2_O as iron precursor, 0.25 mL of propionic acid and 0.5 mL of sulfuric acid in a planetary ball mill (Retsch PM-1000) at 350 rpm for 15 min, employing a 125 mL reaction chamber and eighteen 10 mm stainless steel balls (Pineda et al., 2012). Subsequently the obtained composite was heated up slowly to 800°C for 8 h using an extractor to remove the possible gases formed during the calcination process.

### Preparation of FE-SBA-HSO_3_^BM−IM^ nanocatalysts

The Fe-SBA-HS${\text{O}}_{3}^{\text{IM}}$ catalyst was prepared by a two-step protocol. Firstly, 0.5 g of SBA-15, 1.34 g of Fe(NO_3_)_3_·9H2O and 0.25 mL of propionic acid were milled in a planetary ball mill (Retsch PM-1000) at 350 rpm for 15 min. The obtained material was calcined at 600°C for 8 h. Secondly, 10 mL of sulfuric acid was added drop wise to 1 g of the obtained Fe-SBA-15. After filtration, the solid was washed with distilled water, was heated up slowly to 800°C for 8 h using an extractor to remove the possible gases formed during the calcination process and dried overnight.

### Catalyst characterization

Low-angle XRD pattern were recorded on a Bruker D8 Discover diffractometer equipped with a goniometer Bragg Brentano θ/θ of high precision, and coupled to a Cu X-ray tube. The surface area and pore volume were calculated from N_2_ adsorption–desorption isotherms at liquid nitrogen temperature (77 K) in a Micromeritics ASAP 2000 instrument. The samples were previously degassed for 24 h at 130°C at vacuum conditions (*p* < 10^−2^ Pa).

TEM analysis was performed in the FEI Tecnai G2 system, integrated to a charge coupling device camera. To the preparation, the samples were diluted in ethanol and deposited on a copper grid.

The FT-IR spectra of both nanocatalysts were recorder on an infrared spectrophotometer (ABB MB3000 with Horizon MBTM software), equipped with an ATR PIKE MIRacleTM sampler and a ZnSe window employing 256 scans at a resolution of 16 cm^−1^.

The metal content of the catalysts was obtained by ICP–MS in an Elan DRC-e (PerkinElmer SCIEX) spectrometer. Prior to the analysis, the samples (≈25 mg) were digested using an acid mixture of HF/HNO_3_/HCl 1:1:1. Dilutions were performed with miliQ water up to a maximum of 1% of H${\text{F}}_{2}^{-}$ in acid solution.

Energy dispersive X-ray spectroscopy (EDX) of the obtained materials was carried out using a JEOL JSM-6300 Scanning Microscope with energy-dispersive X-ray analysis (EDX) at 20 kV.

### Oxidation of isoeugenol to vanillin

The oxidation reactions were carried in a carousel system using isoeugenol (0.8 mL, 5 mmol), 30% H_2_O_2_ solution (1.2 mL, 0.04 mmol), 0.10 g of catalyst and acetonitrile (8 mL) as solvent. The reaction mixture was heated at 90°C for 24 h. The progress of the reaction was monitored by withdrawing samples at 20, 40 min, and then every 1 h for 24 h. Samples were analyzed in a HP5890 Series II Gas Chromatograph (60 mL min^−1^ N_2_ carrier flow, 20 psi column top head pressure) using a flame ionization detector (FID). The capillary HP-101 column (25 m × 0.2 mm × 0.2 μm) was employed.

## Results and discussion

**Figure 2:**
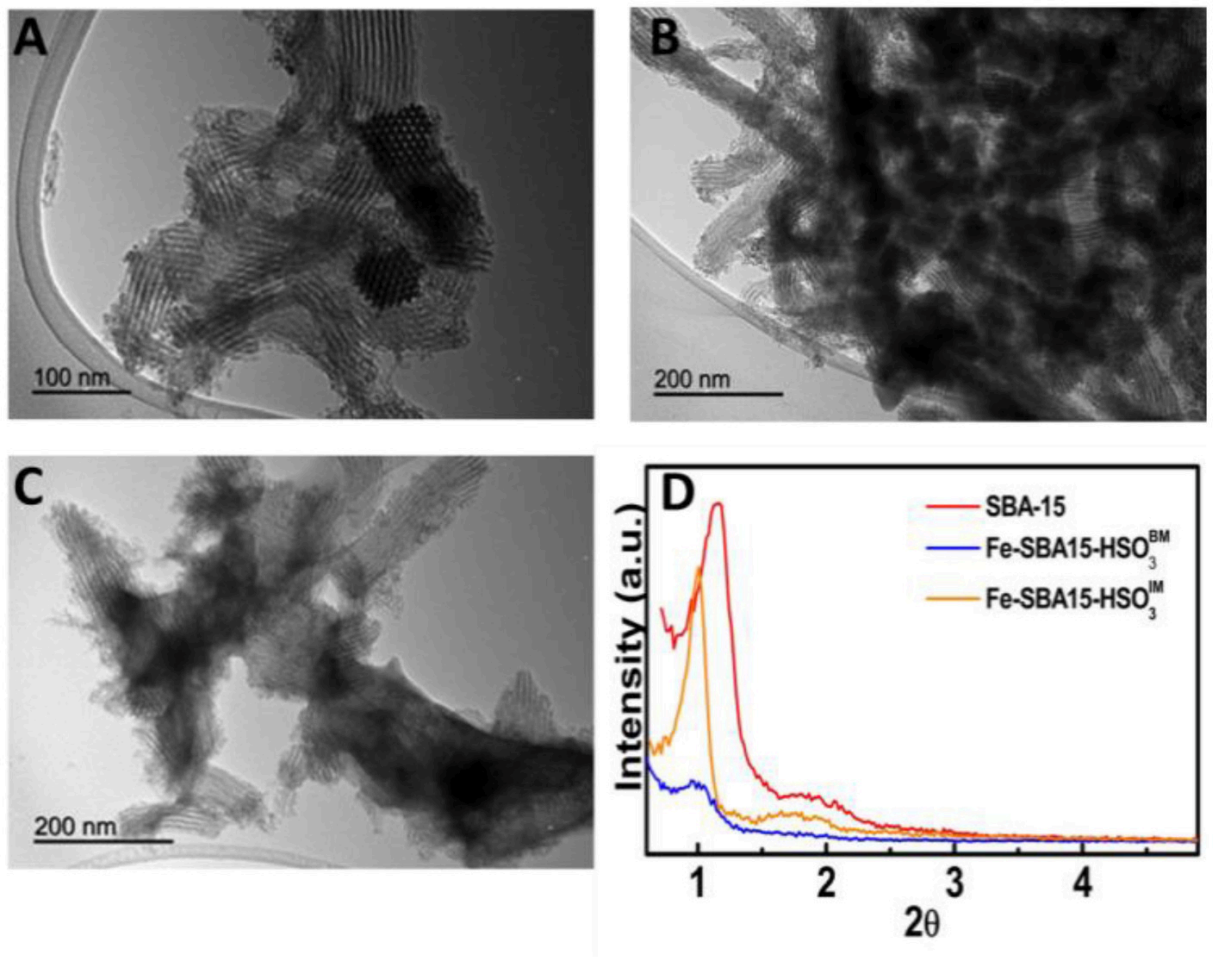
TEM images of **(A)** SBA-15, **(B)** Fe-SBA15-HS${\text{O}}_{3}^{\text{BM}}$ nanocatalyst, **(C)** Fe-SBA15-HS${\text{O}}_{3}^{\text{BM}-\text{IM}}$, and **(D)** small-angle XRD patterns of the nanocatalysts.

The synthesized materials showed a typical diffraction pattern of SBA15-like hexagonal structure which displayed a high intensity (100) peaks at 0.95° and additional order (110) peaks at 1.76° (Figure 2D). The transmission electron microscopy (TEM) images (Figures 2B,C) depicted that Fe containing nanoparticles with size ranging between 4 and 5.7 nm were successfully incorporated inside the nanochannels of SBA-15 (Figure 2A) through the two synthetic routes. However, significant changes in the nanoparticles distribution patterns were observed for the two nanocatalysts. The Fe-SBA15-HS${\text{O}}_{3}^{\text{BM}}$ frameworks have a highly homogeneous and well-dispersed distribution (Figure 2B) which is in agreement with similar materials reported for our group (Pineda et al., 2011); while in the Fe-SBA15-HS${\text{O}}_{3}^{\text{BM}-\text{IM}}$ are highly aggregated. Also, the small shifts to lower diffraction angles of the (100) peaks suggest a slight disordering around the pores while maintaining the hexagonal pore structure upon the synthesis of both nanocatalysts.

**Figure 3:**
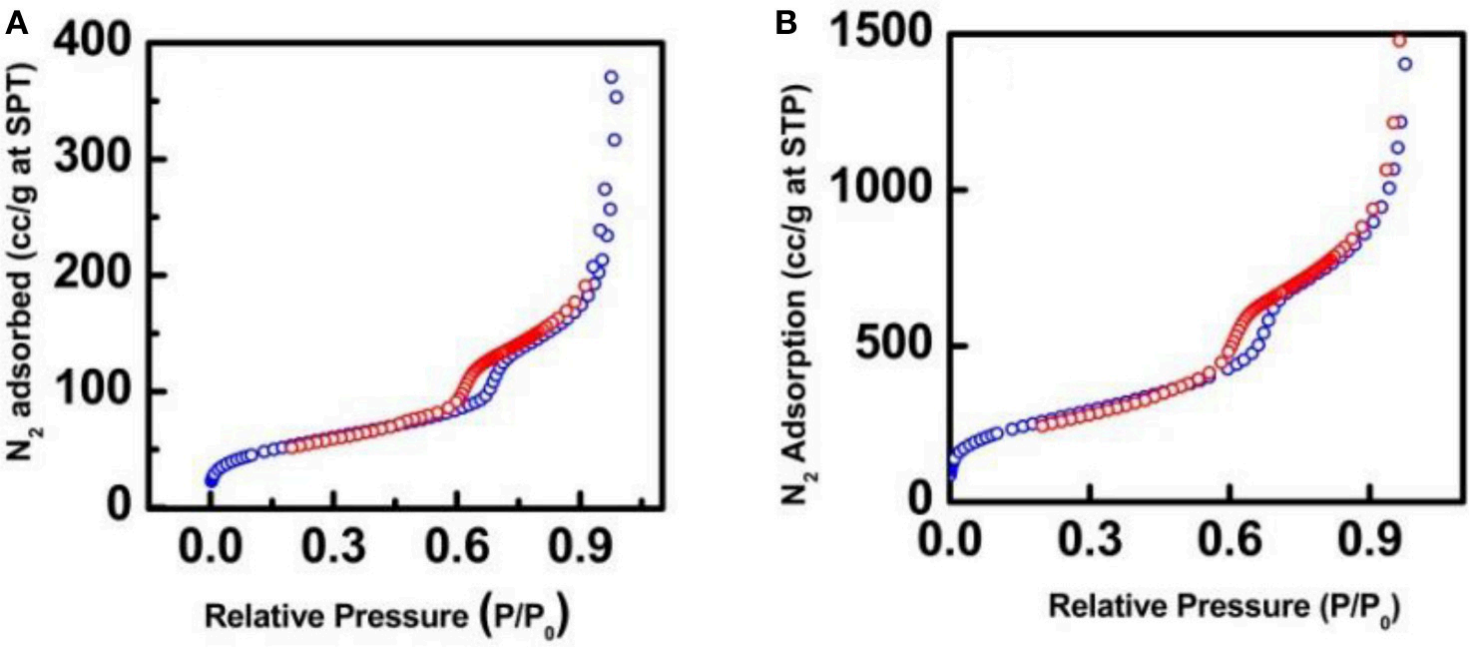
N_2_ adorption-desorption isotherm for **(A)** Fe-SBA15-${\text{HSO}}_{3}^{\text{BM}}$ and **(B)** Fe-SBA15-${\text{HSO}}_{3}^{\text{BM}-\text{IM}}$ nanocatalysts.

The nitrogen adsorption/desorption isotherms were performed to investigate the surface area and pore size properties of the synthesized nanocatalysts. The nanocatalysts possess the typical type IV isotherms and H1 hysteresis loops between 0.6 and 0.75 P/P_0_ (Figure 3), which is a representative behavior of mesoporous materials that contain uniform cylindrical pores (Gao et al., 2007). Interestingly, nitrogen adsorption/desorption experiments displayed a pronounced decay of the surface areas and pore volumes of the nanocatalysts in comparison with the SBA-15 (Table 1). These results can be attributed to the occupation of the SiO_2_ nanochannels for the iron nanoparticles during the synthesis of the nanocatalysts and possible calcination effects for the case of Fe-SBA15-HS${\text{O}}_{3}^{\text{BM}-\text{IM}}$ nanocatalysts (Zhang et al., 2015).

**Table 1:** Textural properties of the synthesized nanocatalysts.

**Material**	**S_BET_^a^ (m^2^ g^−1^)**	**D_BJH_^b^ (nm)**	**V_BJH_^c^ (cm^3^ g^−1^)**
SBA-15	629	5.9	0.75
Fe-SBA15-HS${\text{O}}_{3}^{\text{BM}}$	194	5.3	0.27
Fe-SBA15-${\text{HSO}}_{3}^{\text{BM}-\text{IM}}$	373	5.4	0.35

a *S_BET_, specific surface area was estimated by the Brunauer-Emmett-Teller (BET) equation*.

b *D_BJH_, mean pore size diameter was estimated by the Barret-Joyner-Halenda (BJH) equation*.

c *V_BJH_, pore volume was estimated by the Barret-Joyner-Halenda (BJH) equation*.

**Figure 4:**
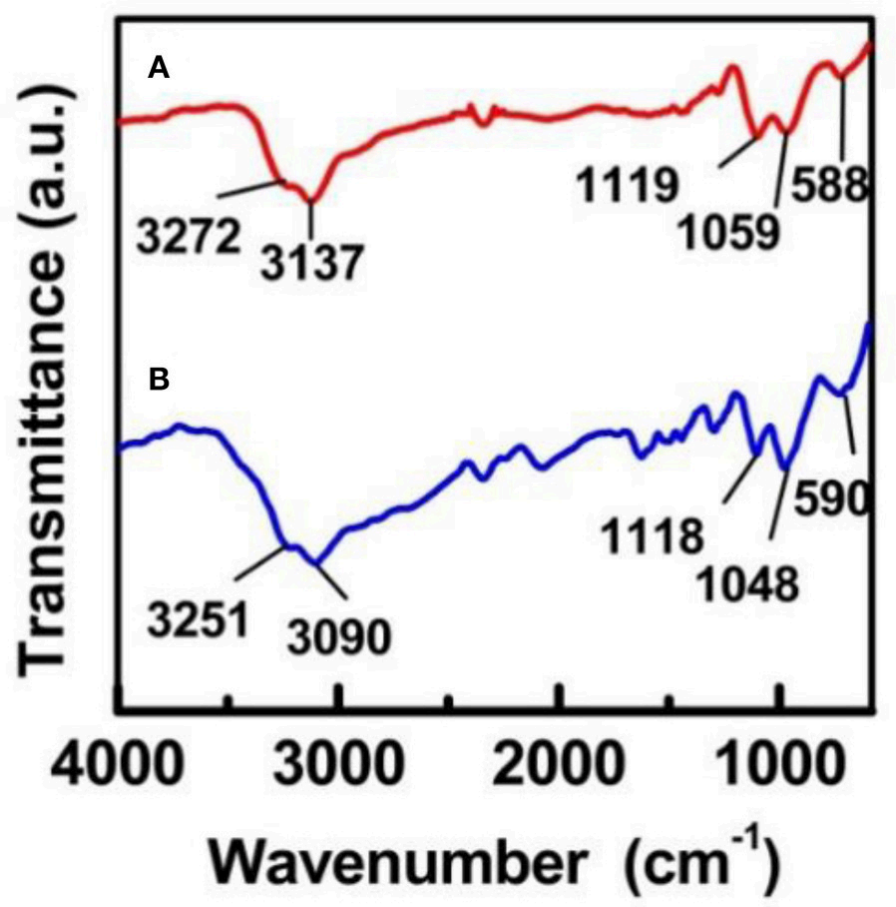
FT-IR spectra of **(A)** Fe-SBA15-${\text{HSO}}_{3}^{\text{BM}}$ and **(B)** Fe-SBA15-HSO_3_^BM−IM^.

Figure 4 depicts the FT-IR results obtained for the Fe-SBA15-${\text{HSO}}_{3}^{\text{BM}}$ and Fe-SBA15-${\text{HSO}}_{3}^{\text{IM}}$ nanocatalysts respectively. In the high frequency regions the spectrum displays a broad band in the region of 2,700–3,600 cm^−1^ which corresponds to -OH stretching absorption of the SO_3_H groups. Bands appeared in the absorption ranges of 1,165–1,248 and 1,007–1,065 cm^−1^ have been ascribed to the O=S=O asymmetric and symmetric stretching modes respectively. Also bands found in the range of 560–608 cm^−1^ were assigned to the S-O stretching mode. All these IR bands strongly support the successful anchorage of the sulfonic groups on both nanocatalysts (Amoozadeh et al., 2016; Kolvari et al., 2016; Veisi et al., 2016).

The elemental composition of the Fe-SBA15-${\text{HSO}}_{3}^{\text{BM}}$ and Fe-SBA15-${\text{HSO}}_{3}^{\text{IM}}$ nanomaterials was determined by both, ICP–MS and EDX, as complementary techniques (Table 2). The ICP-MS measurements of the Fe-SBA15-${\text{HSO}}_{3}^{\text{BM}}$ revealed an iron content of 16 wt%. This result was corroborated by EDX analysis, which displayed 15 and 20 wt% for iron and sulfur, respectively. These values strongly support the successful incorporation of sulfonic groups and iron in the SBA-15 by the applied mechanochemical protocol. Additionally, the elemental content of Fe-SBA15-${\text{HSO}}_{3}^{\text{IM}}$ was determined and compared with the values obtained for Fe-SBA15 before the impregnation process. It was observed a decrease in the iron content after the functionalization of the Fe-SBA15, which have been attributed to the leaching of iron. The high incorporation of iron oxide nanoparticles using the BM method suggests that this approach could represent a new option in the design of novel functionalized nanocatalysts.

**Table 2:** Elemental composition of the nanocatalysts.

	**ICP-MS (wt%)**		**EDX (wt%)**
	**Fe**	**Fe**	**S**
Fe-SBA15	22	21	–
Fe-SBA15-${\text{HSO}}_{3}^{\text{BM}}$	16	15	20
Fe-SBA15-HSO_3_^BM−IM^	0.10	0.25	0.33

**Figure 5:**
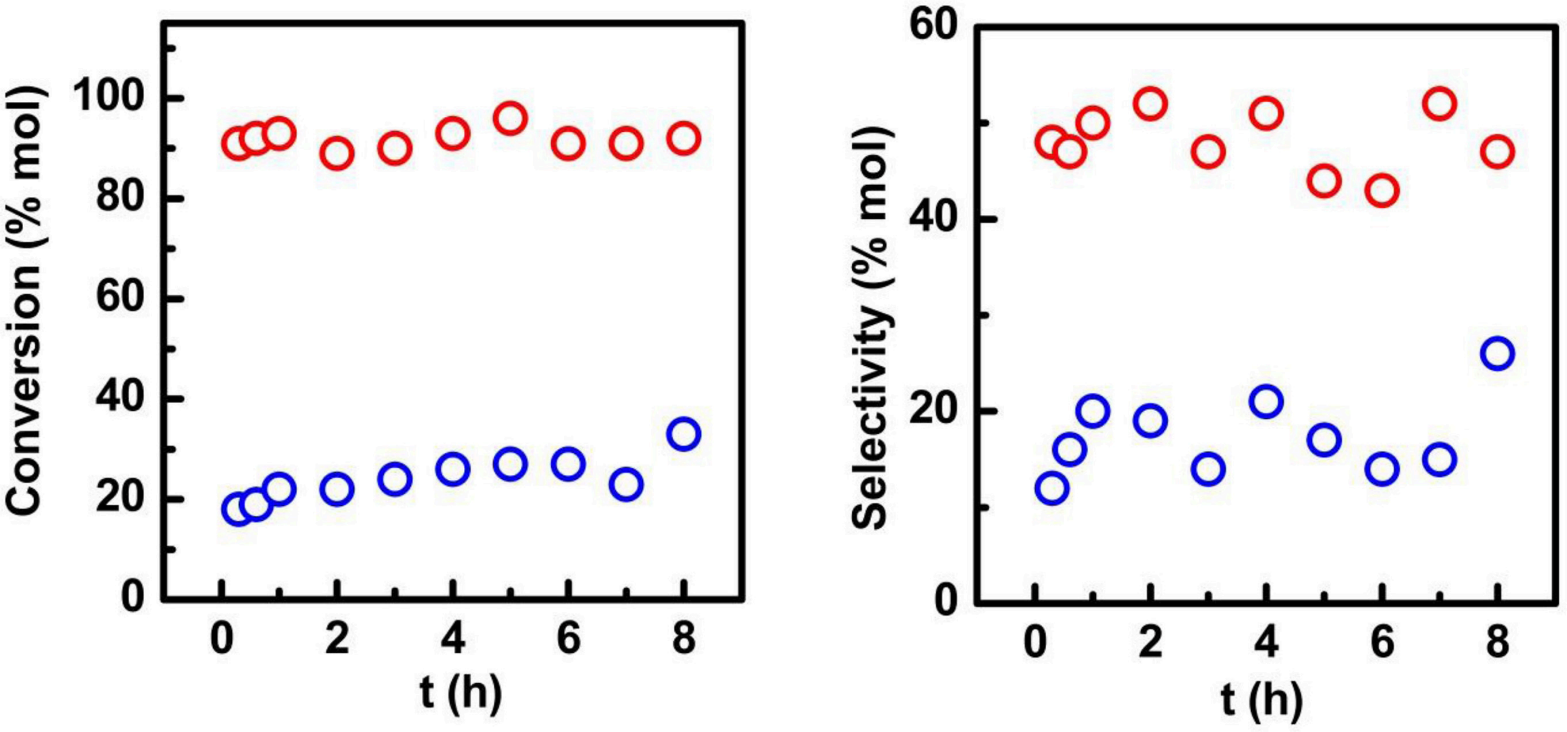
Time dependent conversion and selectivity to vallinin profiles for (red circles) Fe-SBA15-${\text{HSO}}_{3}^{\text{BM}}$ and (blue circles) Fe-SBA15-${\text{HSO}}_{3}^{\text{BM}-\text{IM}}$ nanocatalysts. Reaction conditions: 0.1 g catalyst, 8 mL acetonitrile, 0.8 mL isoeugenol, 1.2 mL H_2_O_2_, *T* = 90°C, time: 1 h.

**Figure 6:**
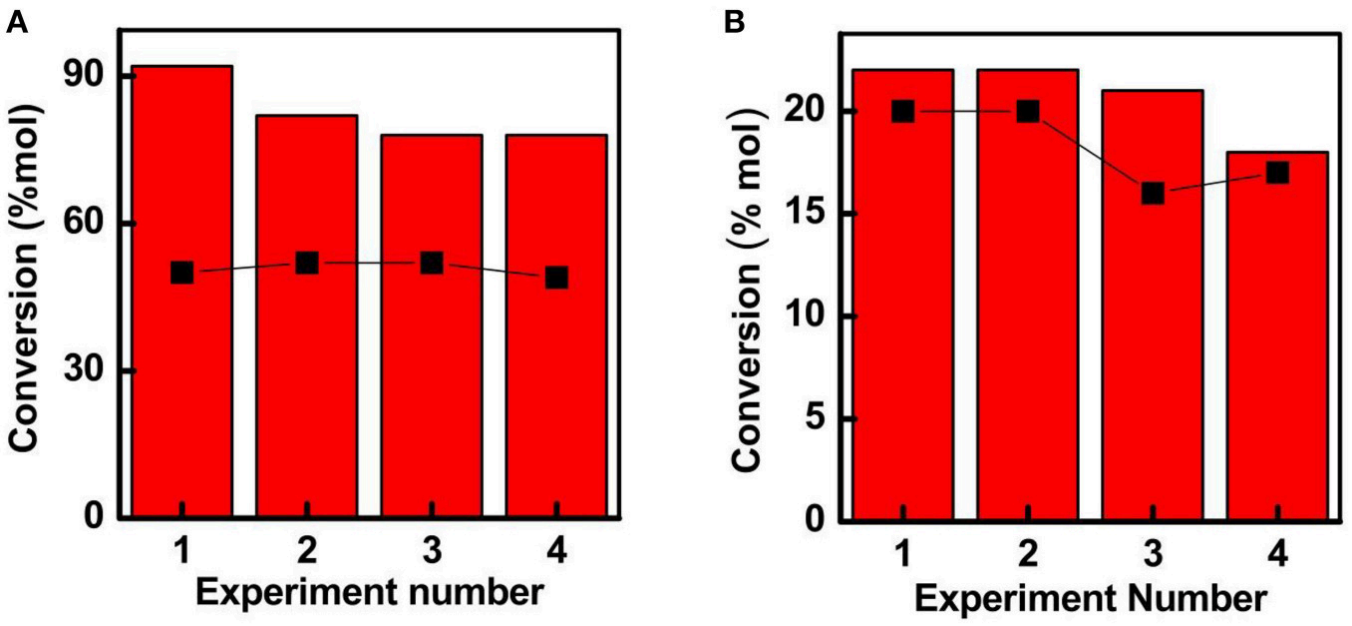
Recycle activities obtained for **(A)** Fe-SBA15-HSO_3_^BM^ and **(B)** Fe-SBA15-${\text{HSO}}_{3}^{\text{BM}-\text{IM}}$ toward the selective oxidation of isoeugenol to vanillin. Selectivity data for both nanocatalysts is inserted by the black squares representation. Reaction conditions: 0.1 g catalyst, 8 mL acetonitrile, 0.8 mL isoeugenol, 1.2 mL H_2_O_2_, *T* = 90°C, time: 1 h.

The catalytic behavior of both nanocatalysts was subsequently investigated to get insights on the effects of their structural variations over the selective oxidation of isoeugenol (Table 3, Figures 5, 6). Low activities were observed in the systems in the absence of the nanocatalysts and in the presence of the SBA-15 (Table 3 entry 1, 2). Under optimized reaction conditions (Table 3, entry 3), the Fe-SBA15-${\text{HSO}}_{3}^{\text{BM}}$ nanocatalyst showed a remarkable higher conversion and selectivity values of 93 and 51% respectively for the selective oxidation of the isoeugenol to vanillin. The lower conversion of the Fe-SBA15-${\text{HSO}}_{3}^{\text{IM}}$ (Table 3 entry 4) could be associated to both, the leaching of the Fe content during the synthesis of the material and the high aggregation of the nanoparticles on specific areas of the mesoporous silica materials which induces the disorganization of the sulfonic acid groups domains.

**Table 3:** Catalytic performances of the nanocatalysts toward the ioseugenol oxidation^a^.

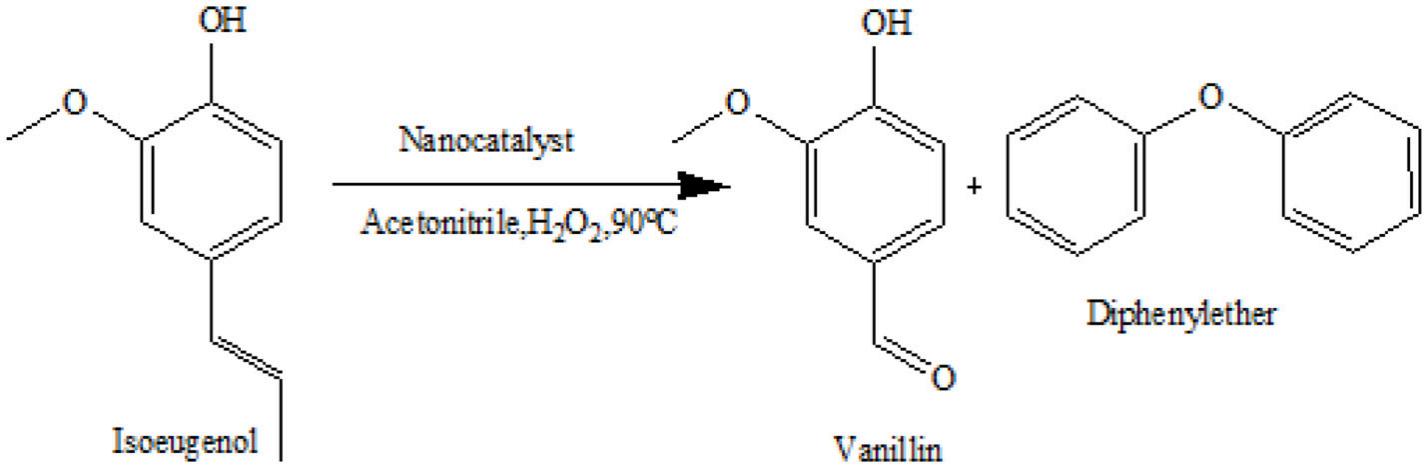						
**Entry**	**Catalyst**	**Time (h)**	**Conversion (% mol)**	**Selectivity (% mol)**		
				**vanillin**	**Diphenyl ether**	**Unidentified products**
1	Blank	1	18	13	64	23
2	15-SBA	1	34	31	49	20
3	FeSBA-15${\text{HSO}}_{3}^{\text{BM}}$	1	93	50	1.8	47
4	FeSBA-15${\text{HSO}}_{3}^{\text{BM}-\text{IM}}$	1	22	20	63	11

a *Reaction conditions: 0.1 g catalyst, 8 mL acetonitrile, 0.8 mL isoeugenol, 1.2 mL H_2_O_2_, T = 90°C, time: 1 h*.

The time dependent conversion and selectivity profiles of the nanocatalysts are shown in the Figure 5. The nanocatalyst synthesized by the mechanochemistry approach displayed a high conversion and selectivity which validates its excellent catalytic performance, while the Fe-SBA15-${\text{HSO}}_{3}^{\text{BM}-\text{IM}}$ nanocatalyst achieved just a 33% after 8 h of reaction.

The reusability of the catalysts constitutes an important parameter to take into account for future applications. At the optimum conditions, the recycling experiments were performed to investigate the stability of the reused catalysts (Figure 6). Noteworthy, the Fe-SBA15-${\text{HSO}}_{3}^{\text{BM}}$ nanocatalyst could be reutilized for at least four catalytic runs without a substantial decay in the activity, suggesting that there is not leaching of the iron nanoparticles during the reaction process. In summary, it have been demonstrated that the iron containing sulfonic acid-functionalized mesoporous silica framework synthesized by a mechanochemical procedure induces a suitable stabilization of the small iron NPs homogeneously distributed inside of the mesoporous framework favoring the enhancement of the catalytic activity of the nanoreactor.

## Conclusions

In summary, we have prepared an efficient heterogeneous nanocatalyst for the selective oxidation of the isougenol to vanillin composed of sulfonic acid functionalized metal mesoporous silica framework using a one-step mechanochemical approach. The catalyst display a framework formed by a highly disperse small iron nanoparticles on the mesoporous silica stabilized by the sulfonic acids domains which induces the enhancement of the catalytic performance of the nanoreactors toward the selective oxidation of the isoeugenol. The work presented here constitutes a significant contribution, not only in the fabrication of effective bifunctional heterogeneous catalysts for the selective oxidation of the isoeugenol to vallinin, but also unraveling the structure,function relationship of the synthesized bifunctional catalysts.

## Author contributions

SO conducted the catalytic reactions and wrote the first draft of the manuscript. AF conducted the synthesis of the catalyst. DR-P performed all the characterization of the materials. AP-S conducted interpretation of the materials and catalytic data. MP-dD revised the manuscript, discussed the results and improved the manuscript for submission. HS and RL conceived the concept of the paper, planned the experiments and revised, rewrote the final manuscript.

### Conflict of interest statement

The authors declare that the research was conducted in the absence of any commercial or financial relationships that could be construed as a potential conflict of interest.
